# Feasibility and effect of integrating tuberculosis screening and detection in postnatal care services: an operations research study

**DOI:** 10.1186/1472-6963-13-99

**Published:** 2013-03-14

**Authors:** Charity Ndwiga, Harriet Birungi, Chi-Chi Undie, Herman Weyenga, Joseph Sitienei

**Affiliations:** 1 Population Council, General Accident Insurance Hse, P. O. Box 17643–00500, Nairobi, Kenya; 2 Division of Leprosy, Tuberculosis and Lung Disease, Ministry of Public Health and Sanitation, P. O. Box 20781–00202, Nairobi, Kenya

**Keywords:** Postnatal care, Tuberculosis screening, Tuberculosis detection

## Abstract

**Background:**

Tuberculosis still remains a major cause of maternal and newborn morbidity and mortality. Integrating tuberculosis screening and detection into postnatal care services ensures prompt and appropriate treatment for affected mothers and their babies. This study therefore examined the feasibility and effect of screening and referral for tuberculosis within postnatal care settings from the perspective of providers.

**Methods:**

This operations research study used a pre- and post-intervention design without a comparison group. The study was implemented between March 2009 and August 2010 in five health facilities located in low-income areas of Nairobi, Kenya, which were suspected to have relatively high prevalence of both tuberculosis and HIV. Descriptive statistics and significance tests were employed to determine changes in the indicators of interest between baseline and endline.

**Results:**

Among the 12,604 postnatal care clients screened, 14 tuberculosis cases were diagnosed. The proportion of clients screened for at least one cardinal sign of tuberculosis rose from 4% to 66%, and 21% of clients were screened for all six tracer signs and symptoms. A comparison of 10 quality of postnatal care and tuberculosis screening components at baseline and endline showed a highly significant effect on all 10 components.

**Conclusions:**

The findings demonstrate that using postnatal care services as a platform for tuberculosis screening and detection is acceptable and feasible. In addition, linking clients identified through screening to further treatment significantly improved. However, the actual number of cases detected was low. A policy debate on whether to link tuberculosis screening with reproductive health services is recommended before full scale-up of this intervention.

## Background

Tuberculosis (TB) remains a major public health problem globally, with an estimated one third (1.86 billion) of the world’s population infected. TB kills more adults than malaria and is one of the leading causes of mortality among women of reproductive age worldwide [1]. Kenya is ranked 13^th^ among the 22 high burden countries that collectively contribute to about 80% of the world TB cases. The disease accounts for over 145,000 discounted life years lost in the country with the situation being worse for women in the reproductive age group [2]. Tuberculosis is the most common HIV-1-related disease and the most frequent cause of mortality in young women in high prevalence regions. Tuberculosis and HIV-1 are independent risk factors for maternal mortality and adverse perinatal outcomes, and in combination have a greater impact on these parameters than their individual effects [3]. Studies have demonstrated that active screening for TB within antenatal care (ANC) setting is beneficial to both the mother and the un born baby. In referral health centres in southern Africa, about one-sixth of all maternal deaths are due to tuberculosis/HIV-1 co-infection [4]. In another study active screening TB was reported by 23. % of HIV-seropositive and 14% of HIV-seronegative women screened during pregnancy. Out of the 3937 women screened active pulmonary TB was diagnosed in 10/1,454 HIV-seropositve women (688 per 100,000) and 5/2,483 HIV-seronegative women (201 per 100,000) [5]. In Kenya 43% of pregnant women attending ANC were screened for TB and 6 out 18911 pregnant women screened tested positive for TB. Further, the study showed that it is feasible and acceptable for providers to screen for TB during pregnancy [6]. The Kenya study findings led to the introduction of TB screening in ANC services in the country. Although TB detection is encouraged among ANC clients within the Maternal and Child Health (MCH) clinics in Kenya, providers in these settings fail to appreciate the principle of continuum of care from pregnancy through to post-natal period.

Moreover, before the study, Kenya’s Ministry of Health (MOH) guidelines on postnatal care (PNC) did not focus on TB detection and its management for mothers and their babies. The recommended package of services only included three scheduled visits whose consultations emphasize the identification of danger signs or diseases that contribute directly to maternal and neonatal death. Counselling for HIV was recommended as part of the standard postnatal care package, but there was little effort to increase detection of TB among HIV-positive mothers and their babies, even though it has been established that at least one out of every eight (13%) HIV-positive patients is co-infected with TB [7]. This lack of integration of TB in PNC implied a missed opportunity for screening for the disease among mothers and their babies, despite it being a public health concern even in the absence of HIV. Utilizing PNC services to screen for TB therefore provides a platform for early case detection and management of the disease.

In order to address this gap, an operations research study was conducted to determine if PNC providers, in addition to providing routine postnatal care services, could also screen and assess the client’s need for TB services and refer suspected cases for management. The study aimed to assess a tuberculosis screening and referral intervention in postnatal care settings with a focus on four key issues: (1) provider knowledge; (2) documenting the feasibility of integrating TB into postnatal care; (3) procedures /protocols in regard to referral mechanisms for PNC TB clients; and (4) evaluating the effect of TB screening, case detection and treatment on PNC clients.

## Methods

### Study area

The study was implemented between March 2009 and August 2010 in five health facilities located in low-income areas of Nairobi, which were suspected to have relatively high prevalence of both HIV and TB. These included Pumwani Maternity Hospital (the largest maternity hospital in East and Central Africa), Mathare North Health Centre, Kayole Health Centre, Langata Health Centre, and Mbagathi District Hospital. Each of these health facilities offers ANC TB services.

### Study design

This was an operations research study involving a pre- and post-intervention design without a comparison group.

### Intervention description

The interventions were implemented over a period of six months from February to July 2010 and involved four major activities:

1. Developing and pretesting materials

2. Improving provider knowledge on TB integration into PNC

3. Reorganizing client flow

4. Support supervision and strengthening data management

#### Developing and pre-testing materials

This activity was informed by a formative assessment and aimed at ensuring that the project used standard materials that had been approved by stakeholders. Three stakeholder meetings were held with to build consensus on the training materials and the content of TB prevention and detection. The materials developed included: (1) a training module on prevention, control, detection and management of TB for the mother and baby during the post-partum period; (2) a Job Aid on TB screening during PNC; and (3) a monthly monitoring data tool for TB screening during PNC.

The training module covered the magnitude of TB, effects and management of TB during the postnatal period, as well as TB and HIV management for mother and the newborn.

The Job Aid included the five cardinal signs and symptoms for TB: persistent cough for three or more weeks with or without blood stained sputum; chest pain; close contact with a person suffering TB; loss of body weight; and intermittent fever and night sweats. The existing PNC and TB registers were modified, through consultations with the Division of Tuberculosis, Leprosy and Lung Disease (DLTLD) and the Division of Reproductive Health (DRH) to capture information on screening for TB during the post-partum period, which was then reported using a monthly data tool.

#### Improving provider knowledge on TB integration into PNC

Ninety-two (92) service providers from the five intervention sites were trained using the new materials, in addition to the MOH-DRH Targeted Postnatal Care Orientation Package. The training was conducted in four groups with each lasting five days. The participants included the members of the District Health Management Team (DHMT), the staff in charge of facilities, and first-line providers working in MCH units and the TB clinics. About two-thirds of the providers who were trained were enrolled or registered nurses/midwives. Six of the trainees were drawn from the TB clinic.

The training involved illustrated lectures for three days during which demonstrations on how to use the TB Job Aid were conducted. These were followed by half-day practical experience at a health facility, where the trainees practiced providing PNC with TB screening as well as the appropriate use of PNC and TB registers. At the end of each workshop, service providers from the same facility developed respective implementation work plans to be followed during support supervision. A standard pre- and post-test exam was administered to measure the knowledge on post-natal care and TB before and after the training. From the results of the pre- and post-test it was evident that learning took place (Table 1).

**Table 1:** Percent pre- and post-test results scores for the four trainings conducted

	**1st training**		**2nd training**		**3rd training**		**4th training**	
**Scores**	**Pre-test**	**Post-test**	**Pre-test**	**Post-test**	**Pre-test**	**Post-test**	**Pre-test**	**Post-test**
Average	74	90	70	83	71	83	75	83
Lowest	40	72	48	60	48	56	48	60
Highest	92	100	96	100	88	96	96	96

#### Re-organization of client-flow

The recommended PNC components include examination of mother and baby to rule out complications, counseling for danger signs, providing information on care for baby and mother, counseling and testing for HIV and family planning (FP). A client flow analysis undertaken during the formative stage showed that although the individual components were being offered daily in all the facilities, they were not available as a comprehensive package under one unit. There was also inadequate infrastructure (room/couch) in almost all of the intervention sites to offer comprehensive postnatal care services. In this context, clients would queue for their babies’ immunization in one unit and again for the postnatal/FP services in another.

The client flow in four of the five health facilities included in the study (Pumwani Maternity Hospital, Mathare North Health Centre, Kayole Health Center and Mbagathi District Hospital) was re-organized to facilitate easier provision of PNC and TB screening services. In the reorganised services, mothers who had queued for baby immunization services in the child welfare clinic (CWC) room were fast-tracked to the PNC services, to avoid queuing again. In Langata Health Centre, a room was identified to serve as a one-stop unit for PNC services.

#### Supportive supervision and strengthening clinic readiness

The Provincial Health Management Teams (PHMTs) and the District Health Management Teams (DHMTs) in the zones where the project sites are located conducted bimonthly supervision visits in the intervention sites in the early phase of the project, to support the implementation of the action plans developed during training. These were later followed by two quarterly support supervision visits by the same team, during which the quality of PNC, TB screening, and data management and record keeping were strengthened. Discussions were also held with the providers to come up with solutions to weaknesses/problems identified during the visit. The MOH-DRH reproductive health supervision tool with an addendum to include TB screening during the postnatal period was used during the support supervision. In addition, the facilities were equipped with TB drugs, TB registers and Cards necessary for integrating TB into postnatal care.

### Evaluation of the intervention

The project was evaluated using a range of data collection methods. Structured interviews were conducted with the staff in charge of clinic at the five intervention facilities to determine availability of infrastructure, equipment and supplies. A nurse working as a research assistant spent half a day at each clinic completing the clinic inventory using an observation checklist. Information was collected on staffing; staff update/training on PNC and TB in the last 24 months; availability of TB screening reagents within the facility; TB drugs; supplies for PNC services (general supplies for examining a postnatal mother); and FP commodities.

Short structured interviews with 37 service providers at baseline and 32 providers at endline were conducted to assess their training, knowledge, and practices regarding the following: post-natal care; TB screening and management; HIV counseling and testing; and family planning during the postnatal care period. The interviews targeted all providers working within MCH units in all the five intervention clinics.

Trained nurses observed and recorded on a standardized checklist all aspects of postnatal care consultations as well as TB screening, including providers’ provision of quality integrated PNC and TB services. Clients were assigned numbers as they entered the MCH unit. The research assistant picked every second client that was visiting the clinic for postnatal care services for up to six clients for every scheduled visit according to the MOH guidelines, that is, the first visit within 48 hours, second visit within 2 weeks, and third visit within 4–6 weeks. Six client-provider interactions were observed for each category at each clinic. A total of 95 observations were conducted at baseline (out of a proposed sample size of 90) while 96 observations were conducted at endline. After each observation, short exit interviews were conducted with each consenting client after the consultation to explore clients’ perspectives on the extent to which integrated PNC and TB services were offered by providers and their overall impression of the services received. All the clients who were observed consented to the exit interviews at both baseline and endline.

All PNC and TB registers in all the five facilities covering the six-month period from February to July 2010 were also reviewed to determine the number of PNC clients, how many of these clients were screened for TB, and the number that was diagnosed with and treated for TB.

### Analysis

Data analysis involved simple descriptive statistics (frequencies, percentages and means) together with the relevant significant tests (of means and proportions) to determine if there are any significant differences in the indicators of interest between baseline and endline measures for the interventions to be considered to have had an impact.

### Ethical considerations

Written informed consent was obtained for all the respondents in the study including service providers and PNC clients. The Population Council’s Institutional Review Board, the Kenya Medical Research Institute, and the National Council of Science and Technology granted ethical and research clearance for the study.

## Results

### Screening for TB among PNC Clients

A comparative analysis of client-provider interaction at baseline and endline demonstrates a significant increase of TB screening among PNC clients (Table 2) –66% of clients were screened for at least one of the symptoms, compared to 4% at baseline. However, less than a quarter of the clients (21%) were screened for all five tracer signs and symptoms for TB see Table 3 below.

**Table 2:** Number of PNC clients suspected and diagnosed with TB

	**Pumwani**	**Kayole**	**Mathare**	**Mbagathi**	**Langata**	**Total**
PNC clients screened	97% (n=9183)	100% (n=2039)	100% (n=862)	87% (n=63)	99% (n=785)	97% (n=12932)
PNC clients suspected to have TB	8 (n= 9050)	3 (n=2039)	2 (n=862)	0 (n=55)	2 (n=779)	15 (n=12,604)
PNC clients with TB positive sputum smear	8*	2	2	0	1	14

*Notes*: TB: tuberculosis; PNC: post-natal care.

*Three of the eight clients diagnosed with TB were also co-infected with HIV.

**Table 3:** Screening for TB among PNC Clients

	**Observations**	
**History asked on symptoms and signs**	**Baseline (N=95) %**	**Endline (N=96) %**
Persistent cough for three or more weeks with or without blood stained sputum	3	64**
Chest pain	2	51**
Close contact with a case of TB	1	44**
Loss of body weight	1	38**
Intermittent fever and night sweats	1	41**
Clients observed to have been asked at least **ONE**	4	66**
Clients observed to have been asked all the **FIVE** signs/symptoms	0	21**
Clients suspected to have TB and referred for TB test	0	3

*Notes:* TB: tuberculosis; PNC: post-natal care; Differences between baseline and end line are statistically significant at: *p<0.05; **p<0.01.

### TB case detection, treatment and referral

A review of PNC service statistics at the five intervention facilities shows that of all the 12,604 PNC clients screened for TB during the intervention period, only 15 (0.11%) were suspected to have TB, based on the screening tool. Fourteen of them had reported persistent cough for two or three weeks, while one had a history loss of body weight. All 14 clients suspected to have Pulmonary TB were immediately referred to the laboratory for sputum specimen collection and test. Twelve of these were confirmed to have TB and referred to TB clinic within the respective facility for treatment. For the client with a history of loss of body weight, the TB diagnosis was confirmed through a chest X-ray. TB/HIV co-infection was detected in only 3 of the 14 clients (see Table 2).

### Effect of integration of TB screening into PNC on quality of care

A comparison of baseline and endline score shows that the project interventions had a highly significant effect on all 10 components of PNC, along with an addition component of ‘TB screening’ (Table 4).

**Table 4:** Quality of care under an integrated PNC/TB package

**Components**	**Baseline**				**Endline**			
	**Visit**_**1**_**(n=38)**	**Visit**_**2**_**(n=36)**	**Visit**_**3**_**(n=22)**	**Total (n=96)**	**Visit**_**1**_**(n=34)**	**Visit**_**2**_**(n=35)**	**Visit**_**3**_**(n=29)**	**Total (n=98)**
History taking (0–8)	0.82	0.94	0.55	0.80	3.79**	3.51**	2.79**	3.4**
Obstetric history taking (0–6)	0.95	1.69	0.95	1.23	4.24**	3.91**	3.1**	3.79**
Counseling on maternal danger signs (0–8)	0.13	0.06	0.00	0.07	2.56**	1.97**	1.39**	2.01**
Counseling on neonatal danger signs discussed (0–5)	0.05	0.19	0.09	0.11	2.12**	2**	1.17**	1.79**
TB screening (0–6)	0.11	0.08	0.05	0.08	3.63**	2.37**	1.07**	2.4**
Observations and physical exams (0–9)	0.50	0.89	0.86	0.73	4.75**	4.14**	3.45*	4.14**
STI/HIV counseling (0–10)	0.58	0.64	0.77	0.65	2.79**	2.43**	1.55*	2.29**
Advice on mother’s self care (0–3)	0.68	0.63	0.23	0.56	1.63**	1.26**	0.83*	1.25**
Counseling on LAM (0–3)	0.42	0.49	0.41	0.44	1.42**	1.6**	0.97*	1.35**
Continuity of care (0–5)	3.13	3.89	3.14	3.42	4.09**	3.97	3.97*	4.01**
Observations and physical exams for baby (0–4)	0.58	0.28	0.41	0.43	2.26**	1.69**	0.86	1.64**

Notes: PNC: Postnatal care; TB: tuberculosis; STI: sexually transmitted infection; LAM: Lactational amenorrhea method; Visit_1_: within 48 hours; Visit_2_: within 2 weeks; Visit_3_: within 4–6 weeks; Differences between baseline and end line are statistically significant at: *p<0.05; **p<0.01.

## Discussion and recommendations

The findings show that provider knowledge of TB screening, detection and PNC improved as a result of the training received. In addition, screening for TB and linking clients suspected to have TB for further investigations and treatment significantly improved. Screening for TB among PNC clients was based on a job aid that included five tracer conditions adapted from a WHO TB screening tool. Full application of the job aid in screening of PNC clients for TB was limited in the sense that not all clients were asked about all the five tracer conditions. In terms of promoting utilization of the screening tool, a much shorter version (including only 2–3 tracer conditions) focusing on commonly asked tracer conditions is recommended as this would equally serve the same purpose. To ensure a TB screening tool that is more sensitive and easy to use, a policy debate validated the modification of the TB screening tool to a shorter checklist that included four screening questions. The screening questions touched on the following: persistent cough for two or more weeks with or without sputum; excessive sweating or night sweats; loss of body weight; and close contact with a person that has TB. The National targeted Postnatal Care Training Package developed by the Division of Reproductive Health and Partners was also revised to include a component of TB screening and management.

Although there is no empirical evidence of how integration of reproductive health/HIV services affects quality of care, there are suggestions in the literature that it may contribute positively or negatively [8-10]. Findings from this study demonstrate, however, that the process of integrating TB screening into PNC positively contributed to the overall quality of PNC. Moreover, this intervention provided an opportunity to re-establish and re-organize PNC services at the study facilities. Concerted efforts to raise the profile of PNC and to increase women’s attendance are recommended to continue to build on these positive outcomes.

It is also important to mention a challenge that the study confronted. Although providers were carrying out screening (as evidenced by the client record reviews), they were inconsistent in recording the number of clients identified in the registry introduced for this purpose. This raises questions about the additional workload that the TB screening process (record-keeping, specifically) may impose on providers. Future screening efforts should ensure that any associated record-keeping processes are as streamlined as possible.

## Conclusions

In summary, the findings demonstrate that it is feasible to use PNC services as a platform for TB screening and case detection. These findings align with evidence from other studies confirming the feasibility of integrating TB screening services into maternal and child health in resource-constrained settings [11]. However, the actual number of cases detected in the present study was low despite the health facilities being situated in areas expected to have high TB prevalence. It would be important, then, for program managers and policy-makers to decide whether incorporating routine TB screening in reproductive health services is justifiable. Strategic considerations around whether to or not to integrate TB screening into reproductive health services may need to be based on good epidemiological data on TB in Kenya. A policy debate on whether to link TB services with reproductive health services is also necessary before full scale-up of this intervention.

## Abbreviations

APHIA: AIDS Population, and Health Integrated Assistance; ANC: Antenatal Clinic; CWC: Child Welfare Clinic; DHMT: District Health Management Team; DLTLD: Division of Leprosy Tuberculosis and Lung Disease; DRH: Division of Reproductive Health; FP: Family Planning; HIV: Human Immuno-deficiency Virus; LAM: Lactational Amenorrhoea Method; MCH: Maternal and Child Health; MOH: Ministry of Health; PNC: Postnatal Care; STI: Sexually Transmitted Infection; TB: Tuberculosis; WHO: World Health Organisation; USAID: United States Agency for International Development

## Competing interests

The authors declare that they have no competing interests.

## Authors’ contributions

CN made substantial contribution in developing the intervention, implementation and the evaluation of the intervention. She also co-developed the first draft of the paper and contributed to the revision of the final version. HB conceptualized the study, oversaw the overall study implementation, and co-developed the first draft of the paper. CU offered a major contribution to the study process by reviewing the intervention package and the study tools, as well as by reviewing and editing the first draft of the paper, and revising the final version. HW and JF provided technical inputs in the conceptualization of the study, the intervention process and a in a policy debate on the study findings that led to the revision of the TB screening checklist. All the authors are aware of the manuscript is being submitted to the journal. All the authors proof read the final manuscript.

## Pre-publication history

The pre-publication history for this paper can be accessed here:

http://www.biomedcentral.com/1472-6963/13/99/prepub
